# Supplementary material for: The Staphylococcus aureus LXG-domain toxins EsxX and SAR0287 do not promote virulence in a zebrafish larval infection model
Source: Microbiology (Reading). 2025 Dec 12;171(12):001636. doi: 10.1099/mic.0.001636 (PMC12699871; doi:10.1099/mic.0.001636)
Supplement: Uncited Supplementary Material 1. [file mic-171-01636-s001.pdf]

The *Staphylococcus aureus* LXG-domain toxins EsxX and SAR0287 do not promote virulence in a zebrafish larval infection model

Fatima Ulhuq[1], Amy K Tooke[1], Chriselle Mendonca[1], Guillermina Casabona[1], Johann Habersetzer[2], Yaping Yang[1], Margarida C. Gomes[3,4], Felicity Alcock[1], Serge Mostowy[3] and Tracy Palmer[1*]

SUPPLEMENTARY INFORMATION

Table S1. Plasmids used in this study

Plasmid	Details	Reference/source
pBAD-18-Cm	Expression vector containing the inducible arabinose BAD promoter; CmlR	(1)
pBAD-SAR0287$_{LXG}$	pBAD18-Cm encoding SAR_0287 N-terminal region (aa 1 - 314)	This work
pBAD-SAR0287$_{CT}$	pBAD18-Cm encoding SAR_0287 C-terminal region (aa 315 – 556)	This work
pIMAY-apra-insertion	(*aac(3)-IVa*) under control of *rpsF* promoter, with flanking sequences from intergenic region downstream of *SAPIG0009*	This work
pIMAY	*E. coli/S. aureus* shuttle vector, temperature sensitive, CmlR	(2)
pIMAY-*esxX*	pIMAY carrying the flanking regions of *esxX*	This work
pIMAY-MRSAΔ0287	pIMAY carrying the flanking regions of SAR0287	This work
pIMAY-MRSA252Δ*ess*	pIMAY carrying the flanking regions of *essC3*	This work
pIMAY-GFP	pIMAY carrying the flanking regions between *SAPIG0102* and *SAPIG0103* to integrate *gfp* gene between them	This work

Table S2. Oligonucleotide primers used in this study

Primer	Sequence	construct
Bad_fwd	GGCATGCAAGCTTGGCTG	pBAD-SAR0287$_{LXG}$
Bad_rev	TCTAGAGGATCCCCGGGTAC	pBAD-SAR0287$_{LXG}$
SAR_0287 1-314_fwd	GTACCCGGGGATCCTCTAGAAGGAGGTTTCTAGTTATGGGGTACAAAGTTGATATG	pBAD-SAR0287$_{LXG}$
SAR_0287 1-314_rev	AACAGCCAAGCTTGCATGCCTTATGCCATTTTTACTGCATTTTTTATTTTAATATTAC	pBAD-SAR0287$_{LXG}$
CM18	GCGCTCTAGACAGAGGAGGAGCCATGAGTGAATTTGCCCGTAATAATC	pBAD-SAR0287$_{CT}$
CM19	GCGCTCTAGACAGAGGAGGAGCCATGGGGTACAAAGTTGATATG	pBAD-SAR0287$_{CT}$
pIMAY_fwd	TTGATATCGAATTCCTGCAG	pIMAY_ΔesxX
pIMAY_rev	ATCGATACCGTCGACCTC	pIMAY_ΔesxX
305-up-500_fwd	TCGAGGTCGACGGTATCGATGATAAGGATGCTGATATAGC	pIMAY_ΔesxX
305-up-500_rev	AAATCTCCTTTTTATACTCCTTTACTCTTTTATATTTATAATTG	pIMAY_ΔesxX
305-down-500bp_fwd	GGGGTAATAAAAAGGAGATTTAAAATGAATAATACTAAG	pIMAY_ΔesxX
305-down-500bp_rev	CTGCAGGAATTCGATATCAATAAACCAAAATGTGTTTTAGTTTTAC	pIMAY_ΔesxX
MGC394	TATCGATAAGCTTGATATCGATTCATGGAATGCTTTAGAAG	pIMAY-apra-ins

MGC401	CTATAGGGCGAATTGGAGCTACCAAATGAAATACCAACAC	pIMAY-apra-ins
MGC311	GCGCGAATTCATTCATGGAATGCTTTAG	pIMAY-apra-ins
MGC312	GAAACTTTCCTCACTATTATACTTTT	pIMAY-apra-ins
MGC313	TAATAGTGAGGAAAGTTTCAAATGAAT	pIMAY-apra-ins
MGC314	GTATTGCACTTTATATTTGCACCTC	pIMAY-apra-ins
MGC315	AAATATAAAGTGCAATACGAATGGCG	pIMAY-apra-ins
MGC316	GTGCATTGGTCAGCCAATCGACTGGCG	pIMAY-apra-ins
MGC317	ATTGGCTGACCAATGCACATAACAACA	pIMAY-apra-ins
MGC318	GCGCGAGCTCACCAAATGAAATACCAAC	pIMAY-apra-ins
SAR287-A1	CATGGAGCTCTCAATCAGCTCTATCTAATTATGAAAAC	pIMAY_ΔSAR0287
SAR287-A2	GGATAAGAAAGGGGCGTACCCCATAATAAATTTCCC	pIMAY_ΔSAR0287
SAR287-B1	ATGGGGTACGGATAAGAAAGGGGC	pIMAY_ΔSAR0287
SAR287-B2	GCGCGGTACCATATGATCTAACCAGCAATAAAT	pIMAY_ΔSAR0287
SAR287-outfor	CCAAGCCTCTGTCAGCAAAG	pIMAY_ΔSAR0287
SAR287-outrev	TTGGATATATATCTTTGTCCATG	pIMAY_ΔSAR0287
SAR279-301-A	CATGGAATTCAATGTGCGTATACTGACCAC	pIMAY_ΔSAR0287
SAR279-301-B	AGGTTTCTAGTTATGGCAATGAGCGACTTATCATAA	pIMAY_ΔSAR0287
SAR279-301-C	AATATACGATGTTTATGATAAGTCGCTCATTGCCATAAC	pIMAY-ess-M252
SAR279-301-D	CATGGAATTCCATAAAACGTTGTCTACTGG	pIMAY-ess-M252
MGC402	TATCGATAAGCTTGATATCGGACCTGACGTCGCTGCCG	pIMAY-GFP-ins
MGC403	CAATCGCGATCCAAAAAGTCTTTAACACAAACAAAAAAGGAGGAAAC	pIMAY-GFP-ins
MGC404	GACTTTTTGGATCGCGATTGCATGCCTG	pIMAY-GFP-ins
MGC405	CTAGTTCATATATATCGCGAGCTGCATAAAAAAC	pIMAY-GFP-ins
MGC406	TCGCGATATATATGAACTAGGGTGATTTAAAG	pIMAY-GFP-ins
MGC407	TGGATCCCCCGGGCTGCAGGCAAAATTCGCATTTATAGCTAAAAATAATTTTG	pIMAY-GFP-ins

References

1. Guzman LM, Belin D, Carson MJ, Beckwith J. Tight regulation, modulation, and high-level expression by vectors containing the arabinose PBAD promoter. *J Bacteriol* 1995;177:4121-4130.

2. **Monk IR, Shah IM, Xu M, Tan MW, Foster TJ.** Transforming the untransformable: application of direct transformation to manipulate genetically *Staphylococcus aureus* and *Staphylococcus epidermidis*. *mBio* 2012;3: e00277-11.

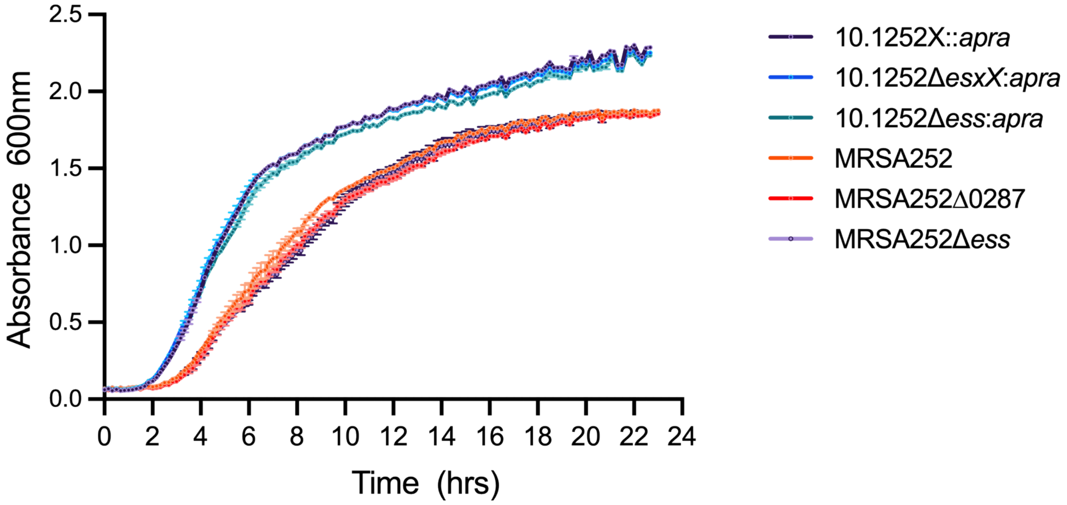

Figure S1. Growth curves for the indicated strains in TSB medium at 33°C.

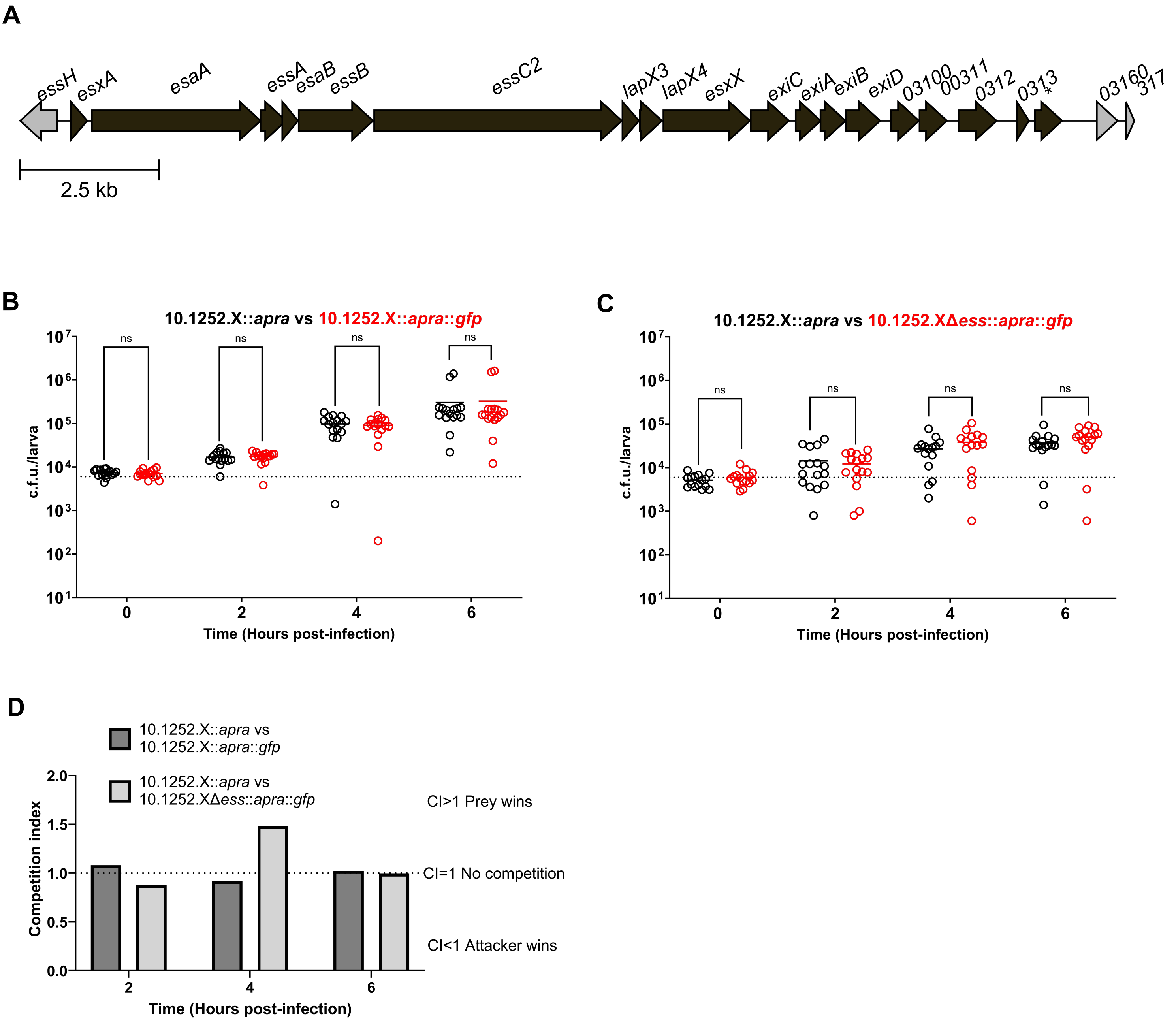

Figure S2. Assessing intrastrain competition in the zebrafish larval hindbrain. A. The T7SS/*ess* locus in 10.1252.X. Genes that are deleted in strain 10.1252.XΔ*ess* are shown in black. *indicates pseudogene. B and C. A 1:1 ratio of the indicated strains (each at 6000 CFU) was injected into the hindbrain ventricle of 3 dpf zebrafish larvae. B. 10.1252.X::*apra* (black) vs 10.1252.X::*apra::gfp* (red), n = 16 total larvae per timepoint, 2 biological replicates. C. 10.1252.X::*apra* (black) vs 10.1252.XΔ*essC*::*apra::gfp* (red), n = 15-16 total larvae per timepoint, 2 biological replicates. At each timepoint larvae were sampled, homogenised and serial dilutions plated on TSA to determine CFU per larva. Circles represent individual larvae, horizontal bars represent the mean. Dotted lines indicate 6000 CFU (target dose). Multiple unpaired t-test with Welch correction. D. Competition indexes at 2, 4 and 6 hpi, ns, not significant.
